# The Clinical Relevance of p16 and p53 Status in Patients with Squamous Cell Carcinoma of the Vulva

**DOI:** 10.1155/2020/3739075

**Published:** 2020-03-24

**Authors:** Ellen L. Barlow, Neil Lambie, Mark W. Donoghoe, Zin Naing, Neville F. Hacker

**Affiliations:** ^1^Gynaecological Cancer Centre, The Royal Hospital for Women, Sydney, NSW, Australia; ^2^Anatomical Pathology, NSW Health Pathology, Prince of Wales Hospital Sydney, Sydney, NSW, Australia; ^3^Anatomical Pathology, Canterbury Health Laboratories, Christchurch, New Zealand; ^4^Mark Wainwright Analytical Centre, University of New South Wales, Sydney, NSW, Australia; ^5^Serology and Virology Division, Microbiology Department, NSW Health Pathology, Prince of Wales Hospital, Sydney, NSW, Australia; ^6^School of Women's & Children's Health, University of New South Wales, Sydney, NSW, Australia

## Abstract

**Objective:**

To investigate the prognostic significance of HPV status in vulvar squamous cell carcinomas (VSCC) and to determine whether preoperative determination of p16 or p53 status would have clinical relevance.

**Methods:**

Patients treated for VSCC at a tertiary hospital in Sydney, Australia, from 2002 to 2014, were retrospectively evaluated (*n* = 119). Histological specimens were stained for p53 and p16 expression, and HPV status was determined by PCR detection of HPV DNA.

**Results:**

HPV DNA was detected in 19%, p16 expression in 53%, and p53 expression in 37% of patients. Kaplan–Meier survival estimates indicated that p16/HPV-positive patients had superior five-year disease-free survival (76% versus 42%, resp., *p* = 0.004) and disease-specific survival (DSS) (89% versus 75% resp., *p* = 0.05) than p53-positive patients. In univariate analysis, nodal metastases (*p* < 0.001), tumor size >4 cm (*p* = 0.03), and perineural invasion (*p* = 0.05) were associated with an increased risk of disease progression and p16 expression with a decreased risk (*p* = 0.03). In multivariable analysis, only nodal metastases remained independent for risk of disease progression (*p* = 0.01). For DSS, lymph node metastases (*p* < 0.001) and tumor size (*p* = 0.008) remained independently prognostic.

**Conclusion:**

The p16/HPV and p53 status of VSCC allows separation of patients into two distinct clinicopathological groups, although 10% of patients fall into a third group which is HPV, p16, and p53 negative. p16 status was not independently prognostic in multivariable analysis. Treatment decisions should continue to be based on clinical indicators rather than p16 or p53 status.

## 1. Introduction

Two subtypes of VSCC have previously been defined. The more common keratinising type typically occurs in older women, is generally associated with lichen sclerosus and/or differentiated vulvar intraepithelial neoplasia (dVIN) [1], and is often associated with p53 tumor suppressor gene mutations [2, 3]. The other subtype is more common in younger women and primarily associated with human papilloma virus (HPV) infection, and a common precursor is usual-type vulvar intraepithelial neoplasia (uVIN) of the basaloid or warty type [4, 5].

p53 is a tumor suppressor gene which is involved in maintaining genomic integrity by controlling cell cycle progression or inducing apoptosis. About 50% of primary human cancers carry mutations in this gene [6]. The tumor-suppressive activity of p53 has been attributed to its ability to regulate the transcription of many different genes in response to a range of stress signals [7]. Some viral oncogenes, such as the HPV viral oncogene E6, have been shown to cause p53 to be functionally inactive. This causes deregulated expression of many genes which p53 orchestrates, such as those involved in apoptosis, DNA stability, and cell proliferation [8].

Expression of the cyclin-dependent kinase inhibitor p16INK4A (p16) correlates closely with the presence of high-risk HPV types, and overexpression of p16 is a surrogate marker for HPV-driven neoplasia [9, 10]. The increase in p16 protein production is mainly related to elevated transcription, which is mediated by the high-risk HPV-encoded oncoprotein E7. The latter functionally inactivates the retinoblastoma protein (RB), releasing p16 from negative feedback control [11].

The prognostic significance of HPV DNA, p16 expression, and p53 expression in patients with squamous vulvar carcinomas is controversial. Some authors have suggested that these markers are not independent prognostic factors [12–15], while others have postulated that surgical aggressiveness could be modified depending on the presence or absence of HPV DNA and/or p16 immunohistochemistry [16, 17].

In oropharyngeal squamous cancers, there is a consensus that HPV-positive cancers are associated with a better prognosis and are more sensitive to radiation therapy [18]. This is true also for anal cancers [19].

The main aims of the current study were to further investigate the independent prognostic significance of HPV status in vulvar squamous cell carcinomas and to clarify whether preoperative determination of p16 or p53 status by immunohistochemistry would have any clinical relevance. A secondary aim was to evaluate clinicopathological variables associated with p16 and p53 status.

## 2. Materials and Methods

Ethics approval was obtained from the South Eastern Sydney Local Health District Human Research Ethics Committee (Reference number: 15/151(LNR/POWH/311)). Consecutive patients treated primarily for squamous cell carcinoma of the vulva at the Royal Hospital for Women, Sydney, between February 2002 and February 2014, were included in the study (*n* = 119). Demographic, clinical, surgical, histopathological, 2009 FIGO staging, and outcome data were retrospectively extracted from the medical records. The patients were followed up until death, or until 30/4/2019. All hematoxylin and eosin slides were reviewed by one of the authors (NL), and PCR detection of HPV DNA was performed by another author (ZN).

## 3. Immunohistochemistry

Each invasive carcinoma was stained for p53 (Leica Microsystems, Novocastra reagents) and p16 (Ventana Medical Systems, Roche Diagnostics) on a Leica Bond 111 platform. The staining was interpreted by a gynecologic pathologist (NL) as “positive” or “negative.” To be interpreted as “positive” (indicating a p53 mutation), p53 staining needed to show definite, usually strong, staining in almost all tumor cell nuclei, with a good positive control. A variable, patchy positive pattern of staining was interpreted as the wild-type pattern (“negative”). For p16, a positive pattern was block-like positive nuclear, ± cytoplasmic staining in virtually all tumor cells. Variable and/or patchy positive staining was interpreted as negative.

In almost all cases, the staining pattern for p53 and p16 was clearly positive or negative. There were no cases with a complete negative (null staining) pattern of p53 staining (which would also be indicative of a p53 mutation) in this series.

## 4. HPV DNA Sample Processing and Nucleic Acid Extraction

Formalin-fixed paraffin-embedded tissue specimens were processed for total nucleic acid extraction using the MagNA Pure 96 System (Roche). Firstly, paraffin-embedded tissue blocks were cut into 10 × 3-micron sections (30 microns total). A new microtome blade was used each time to section a new tissue block to avoid cross-contamination between different samples. Tissue sections were then subjected to xylene treatment (800 *μ*l xylene, Sigma-Aldrich) to dissolve paraffin from the tissue. Tissue sections were pelleted by centrifugation at 16,000 ×g to remove xylene waste and then washed using 800 *μ*l of 100% ethanol (Sigma-Aldrich). Following centrifugation and removal of ethanol supernatant, tissue pellets were air-dried for 10 minutes and then digested using 160 *μ*l MagNA Pure 96 DNA Tissue Lysis Buffer (Roche) and 40 *μ*l Proteinase K (Siemens), with an overnight incubation at 55°C. Subsequently, total nucleic acid was extracted from digested tissue *μ*l preparations (200 *μ*l) using the MagNA Pure 96 DNA and Viral NA Small Volume Kit (Roche), with an elution volume of 100 *μ*l. Extracts were stored at −20°C before testing for HPV DNA.

## 5. PCR Detection of Human Papillomavirus (HPV)

PCR detection of HPV DNA was performed using My11 (5′-GCACAGGGYCAYAAYAATGG-3′) and GP6+ (5′-AATCATATTCCTCMMCATGTC-3′) primers, targeting the conserved L1 region of the HPV genome [20, 21]. These primers were kindly provided by Noel Whitaker (School of Biotechnology and Biomolecular Sciences, University of New South Wales, Sydney), and they can detect high risk HPV, as well as low-risk subtypes as described previously [20, 21]. Template nucleic acid (10.5 *μ*l) was added to a 14.5 *μ*l reaction mixture containing 12.5 *μ*l of 2 × MyTaq™ Red Mix (Bioline) and 0.4 *μ*M of each primer (My11 and GP6+). Cycling conditions include initial denaturation at 94°C for 3 min; 50 cycles of 94°C for 30 sec, 55°C for 30 sec, and 72°C for 30 sec, followed by a final extension at 72°C for 3 min. PCR products of 169 bp were expected for HPV-positive specimens and were visualised by gel electrophoresis.

The validity of the entire process (sample processing, total nucleic acid extraction, and HPV PCR amplification) was confirmed by testing known HPV-positive paraffin-embedded tissues (*n* = 2), along with the study samples.

## 6. Statistical Analysis

Descriptive analysis was performed using IBM SPSS Statistics for Windows (version 25) including frequencies and medians to compare p16/HPV and p53 status with clinicopathological variables. Cross tabulations were performed to examine associations between the two groups using Pearson's *χ*^2^ test. If there were less than five observations per cell, a two-tailed Fisher exact test was used. A *p* value of 0.05 or less was considered statistically significant.

Disease-free survival (DFS) was calculated from the date of treatment until the date of disease recurrence. Disease-specific survival (DSS) was calculated from the date of treatment to the date of death from VSCC. All other patients were censored at date of last follow-up, or date of death from another cause, without documented progression of VSCC. Kaplan–Meier estimates of DFS and DSS were calculated within groups determined by p16 and p53 status. Survival comparisons between the groups were performed using the two-sided log-rank test. To determine five-year survival for the Kaplan–Meier analysis, patients' follow-up was censored after 5 years.

Cox proportional hazards models were used in univariate and multivariable analyses to investigate potential prognostic factors for DFS and DSS. These models included eight prognostic variables in addition to p16 and p53 status. Hazard ratios (HR) with 95% confidence intervals (CI) are presented.

## 7. Results

There were 196 patients with vulvar cancer on our database between 2002 and 2014, of whom 119 were included in the study. The remaining 77 patients were excluded because they had nonsquamous histology (*n* = 26), were referred after primary treatment elsewhere (*n* = 12), presented with recurrent disease (*n* = 21), or had insufficient invasive tissue in the pathology blocks to perform immunohistochemical staining (*n* = 18).

HPV testing was performed on 117 samples (2 could not be evaluated) and 22 were HPV positive (19%). p16 and p53 immunohistochemistry were performed on all 119 tissue samples; 63 (53%) were p16 positive and 44 (37%) were p53 positive.

Twelve of the 119 cases (10%) stained negative for p16 and p53 and were HPV negative. In Kaplan–Meier analysis, this group had a disease-specific survival intermediate between the p16 and p53 groups. However, we have excluded them from further analysis as no distinction could be made based on their HPV, p16, or p53 status.

The remaining 107 patients with positive immunohistochemistry were divided into two groups based on their being p16/HPV positive or p53 positive. Of the 22 HPV-positive tumors, 21 were also p16 positive, and one was p53 positive. The HPV/p53-positive tumor was considered more likely not to be HPV-related because of the patient's age (87 years) and the tumor's association with lichen sclerosus. Five cases stained positive for both p16 and p53. Three of these were associated within a background of lichen sclerosus and dVIN and were therefore considered to be HPV-negative cancers. The remaining two were associated with uVIN and were therefore considered to be HPV-positive cancers.

The clinicopathological features of the 107 patients with positive immunohistochemistry are shown in Table 1. There were 101 Caucasian patients, and 6 were of aboriginal descent. Primary surgery was performed on 101 patients (94%), including radical local excision in 87 patients and radical vulvectomy in 14.

Patients with p16-associated tumors were younger (*p* < 0.001) and were more commonly past or present smokers (*p* < 0.001) than those with p53-associated tumors. The p53-associated group had a higher number of patients with perineural invasion (PNI) (*p*=0.001), depth of invasion ≥5 mm (*p*=0.004), positive nodes (*p*=0.011), and higher FIGO stage (*p*=0.02). Patients with p53-positive tumors had a slightly higher incidence of tumor recurrence than the p16-positive group (53% versus 47%, resp.), but this was not statistically significant (*p*=0.07). They were also much more likely to have two or more local recurrences than the p16-associated group (78% versus 22%, resp., *p*=0.03). No significant differences were observed between the groups for tumor size, tumor differentiation, or lymphovascular space invasion (LVSI).

Regarding the primary site of disease, tumors located on the clitoris were more frequently p53-associated (83% versus 17%, resp., *p*=0.003), whereas tumors located on the vulvar vestibule were more often p16-associated (90% versus 10%, *p*=0.04).

Eighty-six patients (80%) had a unilateral or bilateral inguinofemoral lymphadenectomy, or groin node debulking. Of the 20 patients who did not have a groin lymphadenectomy, 11 had Stage 1A, and 8 had early Stage 1B disease. None of these 19 patients developed a groin recurrence with a minimum follow-up of 30 months and were regarded as node-negative for analysis. The one remaining patient received primary radiotherapy. She had no palpable nodes and did not have a groin lymphadenectomy. She died of progressive disease within 6 months of diagnosis and her nodal status was recorded as unknown.

Six patients (6%) received primary vulvar radiotherapy, five combined with chemotherapy. Twenty patients (19%) had adjuvant radiotherapy, 2 to the vulva only, 9 to the vulva, groins, and pelvis, and 9 to the groins and pelvis.

The patients were followed up for a median of 72 months (range 3–198 months). At the completion of the study, 64 patients (60%) were without evidence of disease and 43 patients (40%) had died. Of the 43 deaths, 20 patients (19%) died of disease and 23 of other causes (21%). There were 38 recurrences (36%), of which 29 (27%) were local (four concurrent with a groin recurrence), five (5%) isolated groin, and four (4%) distant. Of the 29 vulvar recurrences, 14 (48%) were at a remote site and 15 (52%) were at the primary site (*p*=1.0). Remote site vulvar recurrences occurred in 8/63 patients (13%) with p16-associated cancers and 6/44 (14%) with p53-associated cancers (*p*=0.9). For both primary and remote vulvar sites, the earliest first recurrence in p16 and p53 cancers occurred at 6 and 3 months, respectively, while the latest first recurrences occurred at 118 months and 53 months, respectively.

## 8. Survival Analysis

**Figure 1:**
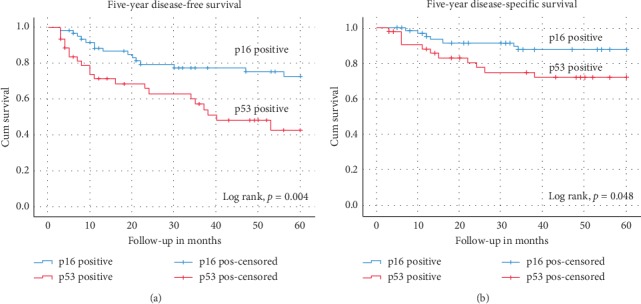
Kaplan–Meier curves for (a) five-year disease-free survival and (b) five-year disease-specific survival stratified by p16-positive and p53-positive groups.

Based on Kaplan–Meier estimates, p16-positive patients had a better five-year DFS than p53-positive patients (76% versus 42%, resp., *p*=0.004) and a better five-year DSS (89% versus 75%, resp., *p*=0.05) (Figures 1(a) and 1(b)).

In the univariate Cox regression analysis for DFS, nodal metastases (*p* < 0.001), PNI (*p*=0.05), and tumor size >4 cm (*p*=0.03) were significantly associated with an increased risk of disease progression, while p16 expression (compared to p53 expression) was associated with a decreased risk (*p*=0.03) (Table 2).

For DSS, tumor size >4 cm (*p* < 0.001), depth of invasion >5 mm (*p*=0.008), nodal metastases (*p* < 0.001), PNI (*p*=0.02), and having had adjuvant radiotherapy (*p*=0.005) were all associated with an increased risk of death (Table 2).

Table 3 shows the multivariable Cox regression model for DFS and DSS. Lymph node metastasis was the only statistically significant independent prognostic factor associated with disease progression (*p*=0.01). For DSS, only tumor size >4 cm (*p*=0.008) and lymph node metastases (*p*=0.001) remained independent prognostic factors in the full model.

## 9. Discussion

Over the last ten years, the reported incidence of HPV DNA in VSCCs has varied between 17% [22] and 59% [4, 5, 23]. In our series, the HPV DNA prevalence rate was 19%, but the prevalence of the surrogate marker p16 was 53%.

Variation in the incidence of HPV infection rates is sometimes attributed to geographical differences [24], differences in the HPV detection methods across studies, and the number of HPV types detected [25]. Our method for detecting HPV DNA, using PCR assays on formalin-fixed paraffin-embedded (FFPE) tissue specimens, is considered the most sensitive, but it has been reported to be potentially impeded by the formalin fixation and paraffin embedding [26]. A recent German study showed a 53% decrease in DNA quantity following a second DNA extraction from 46 FFPE tissue blocks stored for a median time of 5.5 years [27]. As our FFPE samples were stored for a median of 7.6 years (range 2–14 years), this prolonged storage presumably contributed to the relatively low incidence of HPV DNA in our specimens. It also suggests that using PCR assays on FFPE tissue blocks to determine HPV DNA status lacks sensitivity, unless performed on relatively recent tissue blocks.

We used p16 expression to more accurately classify our HPV-related cancers because p16 is strongly overexpressed (without tumor suppressive action) in the presence of high-risk HPV infection due to the functional inactivation of the retinoblastoma protein RB by the HPV-encoded E7 oncoprotein [9, 11]. It is considered an effective surrogate for determining HPV-associated squamous abnormalities of the lower genital tract [9, 10] and squamous vulvar cancers [28].

Our prevalence of p53 expression was 37%, which is within the published range of 28–78% [15, 17, 23, 29, 30]. Like some other studies [29, 31], we found an inverse association between p53 expression and p16 expression, with only five exceptions, and between p53 and HPV DNA, with only one exception. This was not surprising, because mutation of the p53 gene is mostly seen in vulvar cancers which are unrelated to HPV infection [32].

In our study, 10% of the VSCCs were not associated with either p16/HPV DNA or p53 expression. The mechanism of carcinogenesis in this group is unknown, but several other molecular markers have been identified and correlated with clinical outcome in subsets of patients with VSCC. These include epidermal growth factor receptor (EGFR) [29, 33], the c-KIT proto-oncogene, also known as SCFR or CD117 [34], and NOTCH1 and HRAS mutations [17]. Nooij postulated a third molecular subtype of vulvar cancer which was HPV and p53 negative, but p53 wild type with frequent NOTCH1 mutations. In their series, the recurrence rate for this group was intermediate between their HPV-positive and -negative groups [17]. In our series, the p16- and p53-negative tumors also had an intermediate 5-year DSS of 83%, between the p16- (89%) and p53-positive cases (75%).

Univariate analysis showed distinct clinical and pathological differences between patients with p16- and p53-positive cancers. In accordance with previous studies, patients with p16-positive cancers were significantly younger [15, 16, 22, 29, 31], more commonly smokers [22], had earlier-stage disease [17, 22], had tumors which invaded less deeply [22], and had fewer lymph node metastases [17, 22, 31].

In our study, tumors located on the vulvar vestibule were more commonly p16-associated tumors (90% versus 10%, resp., *p*=0.04). Hinten et al. reported that HPV-related cancers were more commonly located on the perineum. They attributed this to the perineum being potentially more susceptible to microtrauma during sexual intercourse, facilitating entry of HPV into the basal cell layer [22]. Should this hypothesis be true, the vulvar vestibule would also be susceptible to such microtrauma.

Our findings for univariate survival confirmed several long-established clinicopathological factors related to DFS and DSS. When adjusted for all other factors in multivariable analysis, only tumor diameter >4 cm and lymph node metastases remained significantly poor prognostic indicators for DSS, and only the latter for DFS. Lymph node metastases [35] and greater tumor diameter [36] are widely recognised as factors associated with negative outcomes for patients with VSCCs.

Previous studies on the influence of HPV/p16 expression on prognosis for VSCC's have reported contradictory results. Tringler et al. also found that patients with p16-positive vulvar cancers had significantly longer DFS and overall survival (OS) in univariate but not multivariable analysis [37]. Two other studies reported no survival advantage for patients with p16/HPV-positive tumors in either unadjusted or adjusted analysis [14, 15]. By contrast, two recent retrospective series have reported p16/HPV-associated tumors to have better DFS and DSS [16], as well as OS [22] when compared to p16/HPV-independent tumors in both univariate and multivariable analyses.

The reported correlation between p53 expression and prognosis in patients with squamous vulvar cancers is also inconsistent. One early study found p53 overexpression to be significantly associated with a poorer prognosis, but only for patients with Stage III disease [38], while others reported no association [12, 13]. More recent studies have reported patients with HPV-positive cancers to have superior survival compared to patients with p53-positive cancers [17, 29]. A recent meta-analysis reported that patients with p16-positive tumors had a significantly better 5-year OS compared to those with p16-negative tumors and that patients with p53-positive tumors had a significantly lower 5-year OS when compared to those with p53-negative tumors [39].

Recently published data support the concept that p16 positivity may be a good prognostic indicator for radiotherapy response in patients with vulvar cancer [40, 41], as has been shown earlier for HPV-positive oropharyngeal cancers [18]. Our study was not designed to make a definitive comment regarding radiotherapy, and our number of patients receiving radiotherapy was small. However, we observed no advantage in DFS or DSS for patients with p16-positive tumors who had adjuvant radiotherapy in multivariable analysis.

Some authors have postulated that the HPV DNA, p16, and/or p53 status of squamous vulvar cancers could be used to change clinical management. In 2016, Hay et al. initially proposed that p53-positive VSCCs may require more aggressive surgery and adjuvant treatment [23]. McAlpine et al. noted a worse outcome for patients with HPV-negative cancers after the introduction of a more conservative surgical approach and postulated that more conservative surgery may be appropriate for younger patients with HPV-positive VSCCs, while patients with HPV-negative cancers may warrant more radical surgery with wider margins and more frequent surveillance [16]. Nooij et al. also suggested the possibility of more aggressive surgery and more stringent follow-up for patients with HPV-negative tumors [17].

Our results would not support any changes to clinical management based on HPV DNA, p16, or p53 status unless it could be shown to be justified in a prospective, randomised clinical trial. Only tumor diameter >4 cm and lymph node metastases were shown to be independent prognostic factors. In addition, remote site vulvar recurrences occurred with a similar frequency to primary site recurrences, as has been reported previously [42, 43], and will occur regardless of the margin status. With regular surveillance for life, preferably done in conjunction with self-inspection of the vulva with a mirror, recurrences can be diagnosed early and resected or radiated with excellent results [43]. One patient with a p16-positive cancer recurred for the first time at 118 months, although such a “recurrence” would have to be regarded as a new primary.

Our study has the limitations of a retrospective design and the inherent restriction in most vulvar cancer studies of limited patient numbers. The study strengths include the combined determination of HPV DNA, together with immunohistochemistry for the biomarkers p16 and p53, and the consistent patient management over the period of the study. Additionally, the long duration of follow-up (median of 72 months) allowed for accurate recurrence and survival outcomes to be assessed.

## 10. Conclusion

The p16 and p53 status of vulvar squamous carcinomas, as determined by immunohistochemistry, allows separation of patients into two distinct clinicopathological groups, although there is a third group which is both p16 and p53 negative. Univariate analysis demonstrated a lower recurrence rate and better survival for patients with p16-positive tumors, but multivariable analysis did not find evidence to suggest that differentiating between HPV/p16 and p53 status provided independent prognostic information. This may be related to the small number of events for recurrence and death from vulvar cancer, but the status of the groin lymph nodes was the only independent prognostic factor for disease-free survival in this study. In view of these results, clinical management should continue to be based on clinical indicators rather than p16 or p53 status.

## Figures and Tables

**Table 1:** Cohort characteristics and the association of clinicopathological variables with p16 and p53 expression.

Variable	Total no. (%)	p16-positive (%)	p53-positive (%)	*p* value
	*N* = 107	*N* = 63	*N* = 44	
Follow-up (months, median)	72 (range 3–198)	72 (range 5–189)	71 (range 3–198)	
Median age in years	71 (range 36–93)	62 (range 39–89)	76 (range 36–93)	
Age groups				
(i) ≤65 years	47 (43.9%)	37 (79%)	10 (21%)	<0.001
(ii) >65 years	60 (56.1%)	26 (43%)	34 (57%)	
Smoking status				
(i) Never	63 (58.9%)	26 (41%)	37 (59%)	<0.001
(ii) Former/current	44 (41.1%)	37 (84%)	7 (16%)	
FIGO stage, *n* (%)				
(i) I	63 (58.9%)	43 (68%)	20 (32%)	
(ii) II	5 (4.7%)	3 (60%)	2 (40%)	
(iii) III	37 (35.6%)	16 (43%)	21 (57%)	
(iv) IV	2 (1.8%)	1 (50%)	1 (50%)	
(v) Stage I/II versus III/IV				0.024
Nodal status†
(i) Positive	39 (36.4%)	17 (44%)	22 (56%)	0.011
(ii) Negative	67 (63.6%)	46 (67%)	21 (31%)	
LVSI				
(i) Yes	21 (19.6%)	11 (52%)	10 (48%)	0.500
PNI				
(i) Yes	15 (14%)	3 (20%)	12 (80%)	0.001^*β*^
Tumor differentiation				
(i) Well	39 (36.5%)	23 (59%)	16 (41%)	0.988
(ii) Moderate/poor	68 (63.5%)	40 (59%)	28 (41%)	
Depth of invasion—mm				
(i) ≤5 mm	59 (55%)	42 (71%)	17 (29%)	0.004
(ii) >5 mm	48 (45%)	21 (44%)	27 (56%)	
Tumor size—cm				
(i) ≤4 cm	73 (68.2%)	46 (63%)	27 (37%)	0.203
(ii) >4 cm	34 (31.8%)	17 (50%)	17 (50%)	
Lesion location				
(i) Clitoris	12 (11.2%)	2 (17%)	10 (83%)	0.003
(ii) Labium minus	22 (20.6%)	14 (64%)	8 (36%)	0.611
(iii) Labium majus	41 (38.3%)	25 (61%)	16 (39%)	0.728
(iv) Perineum	7 (6.5%)	4 (57%)	3 (43%)	1.000^*β*^
(v) Vulvar vestibule	10 (9.3%)	9 (90%)	1 (10%)	0.044^*β*^
(vi) Multifocal	15 (14%)	9 (60%)	6 (40%)	0.924
Adjuvant radiotherapy‡				
(i) Yes	20 (19.8%)	8 (40%)	12 (60%)	0.048
(ii) No	81 (80.2%)	52 (64%)	29 (36%)	
Recurrence				
(i) Any	38 (35.5%)	18 (47%)	20 (53%)	0.073
(ii) Local	29 (27.1%)	14 (48%)	15 (52%)	0.174
(iii) Regional/distant	13 (12.1%)	5 (38.5%)	8 (61.5%)	0.110
(iv) ≥2 local	9 (8.4%)	2 (22%)	7 (78%)	0.031^*β*^

FIGO: International Federation of Gynecology and Obstetrics; LVSI: lymphovascular space invasion; PNI: perineural invasion. †One p53-positive patient nodal status unknown, ‡6 patients were excluded who had primary radiotherapy. Statistically significant value (*p* < 0.05)—Pearson's Chi-square. ^*β*^Fisher's exact test for cell counts <5.

**Table 2:** Univariate outcome analysis by Cox regression for disease-free survival and disease-specific survival.

Variable	Disease-free survival			Disease-specific survival		
	HR	95% CI	*p* value	HR	95% CI	*p* value
Age >65 years (ref—age ≤65 yrs)	1.62	(0.84–3.10)	0.15	2.32	(0.90–6.05)	0.09
Lesion size >4 cms (ref—≤4 cm)	2.05	(1.07–3.92)	0.03	8.05	(3.10–21.05)	<0.001
Depth of invasion >5 mm (ref—≤5 mm)	1.16	(0.62–2.20)	0.64	3.65	(1.40–9.51)	0.008
Lymph node metastases	3.30	(1.73–6.22)	<0.001	23.34	(5.40–101.12)	<0.001
Perineural invasion	2.23	(1.02–5.00)	0.05	3.20	(1.21–8.26)	0.02
LVSI	1.43	(0.70–3.02)	0.35	1.54	(0.56–4.23)	0.41
Differentiation—mod/poor (ref—well-differentiated)	1.20	(0.61–2.31)	0.62	2.62	(0.90–7.85)	0.09
Adjuvant radiotherapy	2.00	(0.92–3.91)	0.08	3.60	(1.45–8.73)	0.005
P16 positive (ref—p53 positive)	0.50	(0.30–0.95)	0.03	0.51	(0.21–1.24)	0.14

HR: hazard ratio; CI: confidence interval; ref: reference group; LVSI: lymphovascular space invasion.

**Table 3:** Multivariable outcome analysis by Cox regression for disease-free survival and disease-specific survival.

Variable	Disease-free survival			Disease-specific survival		
	HR	95% CI	*p* value	HR	95% CI	*p* value
Age >65 years (ref—age ≤ 65 yrs)	1.20	(0.54–2.50)	0.70	2.12	(0.68–6.61)	0.20
Lesion size >4 cm (ref—≤4 cm)	2.05	(0.91–4.63)	0.08	4.90	(1.52–15.80)	0.008
Depth of invasion >5 mm (ref—≤5 mm)	0.53	(0.24–1.15)	0.11	1.01	(0.34–3.03)	0.98
Lymph node metastases	3.03	(1.25–7.35)	0.01	14.83	(2.92–75.20)	<0.001
Perineural invasion	1.72	(0.70–4.25)	0.24	1.80	(0.64–5.00)	0.27
LVSI	0.84	(0.40–2.00)	0.70	0.61	(0.20–1.83)	0.38
Differentiation—mod/poor (ref—well-differentiated)	1.16	(0.60–2.40)	0.70	1.76	(0.47–6.54)	0.40
Adjuvant radiotherapy	0.64	(0.25–1.65)	0.36	0.53	(0.20–1.55)	0.25
p16 positive (ref—p53-positive)	0.68	(0.31–1.50)	0.33	0.90	(0.31–2.50)	0.80

HR: hazard ratio; CI: confidence interval; ref: reference group; LVSI: lymphovascular space invasion.

## Data Availability

The data used to support the findings of this study have not been made available because they are restricted by the Ethics Committee in order to protect patient confidentiality.
